# Influence of Boron, Tungsten and Molybdenum Modifiers on Zirconia Based Pt Catalyst for Glycerol Valorization

**DOI:** 10.3390/nano9040509

**Published:** 2019-04-02

**Authors:** Manuel Checa, Vicente Montes, Jesús Hidalgo-Carrillo, Alberto Marinas, Francisco J. Urbano

**Affiliations:** 1 Departamento de Ingeniería Química y Química Física, Instituto Universitario de Investigación del Agua, Cambio Climático y Sostenibilidad, Universidad de Extremadura, E-06071 Badajoz, Spain; mcheca@unex.es; 2 Departamento de Química Orgánica, Instituto Universitario de Investigación en Química Fina y Nanoquímica (IUNAN), Universidad de Córdoba, E-14071 Córdoba, Spain; vicente.montes@uco.es (V.M.); qo1urnaf@uco.es (F.J.U.)

**Keywords:** acid zirconia, HPAs, Pt catalysts, glycerol valorization, glycerol hydrogenolysis, catalysts deactivation, catalysts stability

## Abstract

The influence of boron, tungsten and molybdenum modifiers on zirconia-based Pt catalyst was studied for glycerol valorization. Zirconia modified supports were prepared by impregnation of ZrO_2_ with either boric, silicontungstic or phosphomolybdic acids to obtain supports with enhanced Brönsted acidic properties. The modified supports were subsequently impregnated with chloroplatinic acid to obtain Pt-based catalysts. Pt incorporation resulted in the increase in Lewis acidity of the solids, being more significant for the Pt//W/ZrO_2_ catalyst. Reduced Pt catalysts were tested for the liquid-phase glycerol hydrogenolysis, observing a synergistic effect between catalyst acid sites and metal function that proved to be crucial in glycerol hydrogenolysis. The Pt//W/ZrO_2_ catalyst was the most active catalyst in this reaction, being the only leading to 1,3-PDO (45% sel., 160 °C) while Pt//Mo/ZrO_2_ is the best option for 1,2-PDO (49% sel., 180 °C). Reusability studies carried out for Pt//W/ZrO_2_ showed that catalytic activity dropped after the first use, remaining constant for the second and subsequent ones. Selectivity to reaction products also changes during reuses. Therefore, the selectivity to 1,2 PDO increases in the first reuse in detriment to the selectivity to n-propanol whereas the selectivity to 1,3-PDO remains constant along the uses. This behavior could be associated to the lixiviation of W species and/or catalyst fouling during reaction runs.

## 1. Introduction

Nowadays, society demands to industries the development of new environmentally friendly processes and chemicals [1]. Academia and industries have focused their attention on biomass-based chemistry because it is the only raw material able to produce both energy and chemicals [1,2]. One example is biodiesel production as an alternative to fossil fuels. The substitution of petrol-based fuel by renewable raw materials as vegetable oils can considerably reduce their environmental impact [3]. In this line, many countries have built facilities in order to increase their biodiesel production as, for example, European countries whose production represents up to 30% of the global production [4]. Such increment directly affects glycerol markets since biodiesel industries generate 1 kg of bioglycerol (as by-product) per 10 kg of biofuel [4,5,6]. This caused the glycerol saturation of the markets and consequently a price drop of that by-product [7,8,9,10]. The low cost of glycerol, together with the high functionality of this molecule, make it an ideal starting material for several chemical routes [11].

From the glycerol valorization point of view, one of the most important processes is the hydrogenolysis to diols [12,13]. This reaction can lead to different products such as 1-hydroxypropan-2-one (Acetol), 1,2-propanediol (1,2-PDO), 1,3-propanediol (1,3-PDO) or ethylene glycol (EG). However, these products are produced simultaneously thus requiring further separation steps and leading to an increase in the production costs [13,14,15]. 1,2-PDO and Acetol production from glycerol can be an economically important alternative for these compounds, which have several applications for industries as solvents or intermediates for other valuable products or polymers [16,17,18]. On the other hand, 1,3-PDO is a valuable monomer for different polymer synthesis that is traditionally obtained via acrolein selective hydration, however, its production from glycerol in biorefineries being a promising alternative [19]. By using different tools like heterogeneous catalysis, the reaction can be tuned up to yield the desirable product, thus reducing or better avoiding the mentioned separation problems [20,21]. In this line, a wise combination of active metal and acid-base properties of the support can influence the reaction in order to maximize the production of one of the desired products, such as 1,2-PDO, Acetol [22,23,24] or 1,3-PDO [19,22,23,24,25,26].

Most described metals in the literature for glycerol hydrogenolysis are transition metals such as Cu, Ni or Co [8,27], but also some noble metals such as Pd [28], Rh [18] or Pt [29,30,31,32,33] can be used. Among them, Pt is the preferred one since it is able to avoid C2 and C1 products, that is, to avoid C-C breakage [30].

Regarding the support, metals are supported on different solids such as metal oxides [18,25,33,34,35], zeolites [33], carbonates [36] or charcoal [37], just to cite some of them. Among metal oxides, ZrO_2_ is an interesting support since its acid-base properties can be easily tuned up by employing different dopants or modifiers [38,39]. Therefore, for example, borated zirconia exhibits both acid and basic sites [40]. Incorporation of Mo [41] or W [42,43,44,45] have been widely studied for the hydrogenolysis of glycerol, tungsten being described as selective to 1,3 PDO or C-3 mono-alcohols depending on the conditions [46,47]. In fact, Arundhathi and co-workers [48] described a tungsten-based catalyst exhibiting a high yield to 1.3-PDO (66%) at full conversion.

Recently, heteropoly acids (HPAs), both tungsten and molybdenum, have attracted attention for the use as catalyst modifiers for the valorization of glycerol, since they possess unique physical-chemical properties and well-defined Brönsted acidity. Zhu et al. [49] observed that the modification of a Pt/ZrO_2_ catalyst with different HPAs of Mo and W, such as HSiW, HPW or HPMo, increased the catalytic activity and 1,3-PDO selectivity due to the enhanced acidity. This improved acidity is associated to the W and the Mo, and not to the silicon or phosphorus [49].

The purpose of this piece of research is to study the influence of different acidic modifiers incorporated to zirconia and used as support in supported Pt catalysts and its application to the glycerol hydrogenolysis process. Thus, boric (H_3_BO_3_), silicotungstic (H_4_SiW_12_O_40_) and phosphomolibdic (H_3_PMo_12_O_40_) acids were used as modifiers and their influence on activity and selectivity of glycerol hydrogenolysis studied.

## 2. Materials and Methods

### 2.1. Synthesis of the Catalytic Systems

The catalysts were synthesized in three subsequent steps which involves (1) the precipitation of ZrO_2_ as the base support, (2) the modification of this support by impregnation with different acidic modifiers and, finally, (3) the incorporation of Pt into the modified support following the incipient wetness impregnation method.

In order to obtain 40 g of bulk zirconia, 250 mL of zirconyl chloride solution (30% in HCl, Sigma-Aldrich^®^) was dissolved in 1 L of *Milli-Q* water. Then, under mechanical stirring, the pH was increased by adding NH_4_OH with a Syrris^®^ Atlas Syringe Pump (Hertfordshire, UK), in a two-step process: from 1 to 5.7 by adding NH_4_OH 5N and then from 5.7 to 7.6 by using NH_4_OH 0.1N. The zirconia gel was stirred overnight and then filtered and washed repeatedly with *Milli-Q* water until the AgNO_3_ chloride test was negative. The obtained gel was then dried overnight at 110 °C and finally milled, sieved (0.149 mm) and calcined at 300 °C for 6 h, under air flow (30 mL·min^−1^; heating rate: 2 °C·min^−1^). The calcined material was divided into four portions (*ca.* 10 g each). Among them, three portions were impregnated with the acid modifiers, whereas the fourth unmodified zirconia was used as the reference bare support.

Modifiers, such as boric acid (Sigma-Aldrich^®^ Merck KGaA, Darmstadt, Germany), phosphomolibdic acid hydrate (Sigma-Aldrich^®^ Merck KGaA, Darmstadt, Germany) or silicotungstic acid hydrate (Sigma-Aldrich^®^ Merck KGaA, Darmstadt, Germany), were incorporated by impregnation in a M/Zr atomic ratio of 1:10, where M stands for B (boron), W (tungsten) or Mo (molybdenum). The appropriate amount of a 1 M solution of the modifier was placed in a 50 mL round bottom flask together with 10 g of ZrO_2_. The mixture was rotated (150 rpm) at room temperature for 5 h and then the solvent was evaporated at controlled temperature (1 h at 30 °C, 1 h at 50 °C and 80 °C until dryness). The flask was then removed from the rotary evaporator and placed in an oven at 110 °C overnight. The solid was then milled, sieved and calcined at 300 °C for 6 h, in air flow (30 mL·min^−1^). Finally, the calcined supports were again milled, sieved (0.149 mm) and labelled as M/ZrO_2_, where “M” stands for boron (B), molybdenum (Mo) or tungsten (W).

Finally, the incorporation of platinum to the previously synthesized supports was carried out via incipient wet impregnation with chloroplatinic acid (8 wt.%, Sigma-Aldrich^®^ Merck KGaA, Darmstadt, Germany). To obtain a Pt loading of 5 wt.%, the appropriate amount of chloroplatinic acid was introduced in a 50 mL round bottom flask containing 9.5 g of support. The mixture was then rotated for 5 h, the solvent evaporated, and the solid dried, milled, sieved (0.149 mm) and calcined at 300 °C in a similar way to the above stated procedure.

Before catalytic tests, Pt catalysts were reduced in H_2_ flow (30 mL·min^−1^) at 200 °C for 2 h (heating rate 10 °C·min^−1^). Once reduced, the solids were cooled down in H_2_ to room temperature and then purged for 15 min with N_2_ (30 mL·min^−1^). The reduced catalysts were named including the metal, modifier and support, followed by activation (hydrogen) temperature: Pt//M/ZrO_2_-200.

### 2.2. Characterization of the Catalysts

Chemical analysis of metal and Pt-containing samples was carried by inductively coupled plasma with mass spectrometry detection (ICP-MS) at the Central Service for Research Support (SCAI) of the University of Cordoba. Measurements were made on a Perkin-Elmer ELAN DRC-e instrument (Waltham, MA, USA) following dissolution of the sample in a two-step process: 100 mg was treated with 20 mL of the HCl:HNO_3_ (3:1 *v*/*v*) and stirred under mild temperature for 5 min. Then, 5 mL of HF was added to the mixture at 50 °C and stirred until complete dissolution of the solid sample. All solutions were diluted to 100 mL with 3% HNO_3_ before analysis. Calibration plots were performed using Perkin Elmer Pure Plus atomic spectroscopy standards.

X-ray diffraction analysis (XRD) of the samples was carried out on a Bruker D8 Discover A25 diffractometer (Bruker Corporation, Billerica, MA, USA) equipped with a VÅNTEC-500 bidimensional detector equipped with an automatic control and data acquisition system. The instrument used CuKα radiation and a graphite monochromator. The samples were reduced at 200 °C before the analysis.

X-ray photoelectron spectroscopy (XPS) data were carried out at the Central Service for Research Support (SCAI) of the University of Cordoba on a Leibold—Heraeus LHS10 spectrometer (Bad Ragaz, Switzerland) capable of operating down to less than 2 × 10^−9^ Torr, that was equipped with an EA-200MCD hemispherical electron analyzer with a dual X-ray source, using AlKα (hν = 1486.6 eV) at 120 W, at 30 mA, and utilising C (1s) as energy reference (284.6 eV). The spectra were recorded on 4 × 4 mm pellets 0.5 mm thick that were obtained by gently pressing the powdered materials following outgassing to a pressure below about 2 × 10^−8^ Torr at 150 °C in the instrument prechamber to remove chemisorbed volatile species.

Surface areas of the solids were determined from nitrogen adsorption–desorption isotherms obtained at liquid nitrogen temperature on a Micromeritics ASAP-2010 instrument (Micromeritics, Norcross, GA, USA), using the Brunnauer–Emmett–Teller (BET) method. All samples were degassed to 0.1 Pa at 120 °C prior to measurement.

Temperature-programmed reduction (TPR) and chemisorption measurements were carried out on a Micromeritics Autochem II chemisorption analyzer (Micromeritics, Norcross, GA, USA). For TPR analysis, 50 mg of calcined catalyst was placed in the sample holder and pretreated by heating at 300 °C in Ar (40 mL·min^−1^) and cooled down until 50 °C. Then, the sample was analyzed in a 10% H_2_/Ar stream (40 mL·min^−1^) by increasing the temperature from 50 to 600 °C at 10 °C·min^−1^.

For chemisorption analysis, 50 mg of solid was placed in the sample holder and reduced at 200 °C in H_2_ flow (30 mL·min^−1^). Then, the temperature was stabilized at 50 °C and the samples were analyzed by performing loop injections with pure H_2_ each 2 min until constant peak areas were obtained. Calculations were performed by assuming hemispherical platinum metal particles.

Surface acid-base properties of the catalysts reduced at 200 °C were determined from the results obtained in the propan-2-ol transformation test reaction. This process, widely described as a model reaction, can provide valuable information on surface acid-base properties of the catalyst as a function of its products distribution. Thus, surface acid sites yield propene and/or diisopropyl ether while basic or redox properties lead to acetone and/or diacetone alcohol [50,51,52].

The gas-phase reaction was carried out in a stainless steel reactor (1/8 inch OD) that was loaded with 100 mg of catalyst and 1 g of inert SiO_2_. Prior to the reaction, the catalyst was reduced under an H_2_ flow (20 mL·min^−1^) while increasing the temperature until 200 °C (rate, 2 °C·min^−1^). The reaction was started by introducing propan-2-ol by passing a nitrogen flow of 10 mL.min^−1^ through a saturator with propan-2-ol at room temperature. Analyses were carried out on-line by connecting the effluent to a gas chromatograph (HP 5890 series II) equipped with a capillary column Supelcowax-10 (60 m long, 0.25 mm ID, 0.25 μm film thickness).

The above-described experiments were complemented with diffuse reflectance infrared Fourier Transformed (DRIFT) spectroscopic studies of pyridine-adsorbed solids intended to identify the specific type of surface acid sites. Measurements were made with an ABB Bomen MB Series IR spectrophotometer equipped with a SpectraTech environmental chamber including a diffuse reflectance device capable of performing 258 scans at 8 cm^−1^ resolution at an adjustable temperature. Prior to analysis, the reduced catalyst was cleaned by heating at 300 °C for 30 min. The catalyst was then saturated with pyridine under controlled atmosphere and heated to 100, 200 and 300 °C, recording the DRIFT spectra 60 min after the selected temperature was stabilized.

Thermogravimetric and differential thermal analysis (TGA-DTA) analysis of Pt//W/ZrO_2_ spent catalyst was carried out on a Setaram SetSys 12 instrument (SETARAM Instrumentation, Caluire–FRANCE). A total of 20 mg of sample was placed in an alumina crucible for TGA-DTA analysis and heated from 30 to 800 °C at a rate of 10 °C·min^−1^ under synthetic air (40 mL·min^−1^) in order to measure weight loss, heat flow and derivative weight loss.

### 2.3. Reactivity Tests

The reduced catalysts were tested for the catalytic hydrogenolysis of glycerol in a high pressure Berghof reactor (operation limit conditions: 250 °C, 100 bar and 80 mL of volume). The reactor was filled with 10 mL of an aqueous 1.36 M solution of glycerol (99%, Sigma-Aldrich^®^) and 100 mg of freshly reduced catalyst. Then, the reactor was closed, purged with H_2_ for 1 min and filled at room temperature with 6 bar of H_2_. Then, the reaction temperature was set (160, 180 or 200 °C) and the system allowed for stabilization for 1 h. The reaction was started by switching on the stirring (1000 rpm) and stopped at a selected time by introducing the reaction on an ice bath. Additionally, some reactions were carried with acetol (90%, Sigma-Aldrich^®^ Merck KGaA, Darmstadt, Germany) or 1,2-PDO (Sigma-Aldrich® Merck KGaA, Darmstadt, Germany) 0.5 and 1 M, respectively, as starting substrates. Blank experiments were carried out in the absence of a catalyst and with bare supports. The gas-phase evolved was analyzed on line by coupling the reactor outlet to a GC-FID (Agilent^®^ 6890, Santa Clara, CA, USA) while the reactor was depressurized. The liquid phase was homogenized, filtered through a syringe filter (0.45 µm) and analyzed off-line by GC-FID (Agilent^®^ 7890, Supelco^®^ Nukol column).

The carbon mass balance was found to be >96%, considering both gas and liquid phases. The conversion of glycerol and the selectivity to each product are defined as follows:

(1) $$\%Conversion=\frac{molGly_{0}-molGly_{f}}{molGly_{0}}\times 100,$$

(2) $$\%Selectivity=\frac{n_{i}\times M_{i}}{3\times (M_{Gly}{}_{{}_{0}}-M_{Gly}{}_{{}_{f}})}\times 100,$$

(3) $$\%Yield=\frac{Conversion\times Selectivity}{100},$$

where *n_i_* is the number of carbon atoms in the compound “*i*” and *M_i_* is the molar concentration of the compound.

## 3. Results and Discussion

### 3.1. Textural and Structural Characterization of the Solids

Supports and supported Pt catalysts were thoroughly characterized from a textural, structural and chemical point of view by a wide number of techniques. Table 1 presents the main features concerning Pt catalysts. Bare ZrO_2_ presented a BET surface area of 126 m^2^·g^−1^ while its modification led to a decrease in surface area. The incorporation of platinum led to a decrease in the specific surface of ZrO_2_ to 109 m^2^·g^−1^, while the incorporation of both modifiers and platinum, resulted in a deeper decrease in surface area, the lowest values corresponding to Pt//Mo/ZrO_2_ and Pt//W/ZrO_2_, 86 m^2^·g^−1^ in both cases.

The platinum content in the final catalysts was determined by ICP-MS (Table 1). ICP-MS shows that Pt incorporation is close to the nominal value, with 4.6 wt.% Pt loading for all catalysts, except for Pt/ZrO_2_ with 4.8 wt.%.

Regarding the modifiers loading, Boron loading (11 at.%) was slightly higher than the nominal value (10 at.%), while in Pt//W/ZrO_2_ and Pt//Mo/ZrO_2_ catalysts the amount of modifier determined by ICP-MS was 8.3 and 7.0 respectively, slightly lower than the nominal amount.

XPS analysis revealed that, in all cases, the amount of chlorine in the 10%–12% range. This species not only comes from the zirconium oxychloride but also from the platinum precursor. The O/Zr ratio is close to the stoichiometric value of ZrO_2_ (2). Moreover, the platinum atomic % is similar in all cases, except for Pt//Mo/ZrO_2_. The larger platinum particle size (as determined by hydrogen chemisorption) for this sample as compared to the others could account for that.

**Figure 1:**
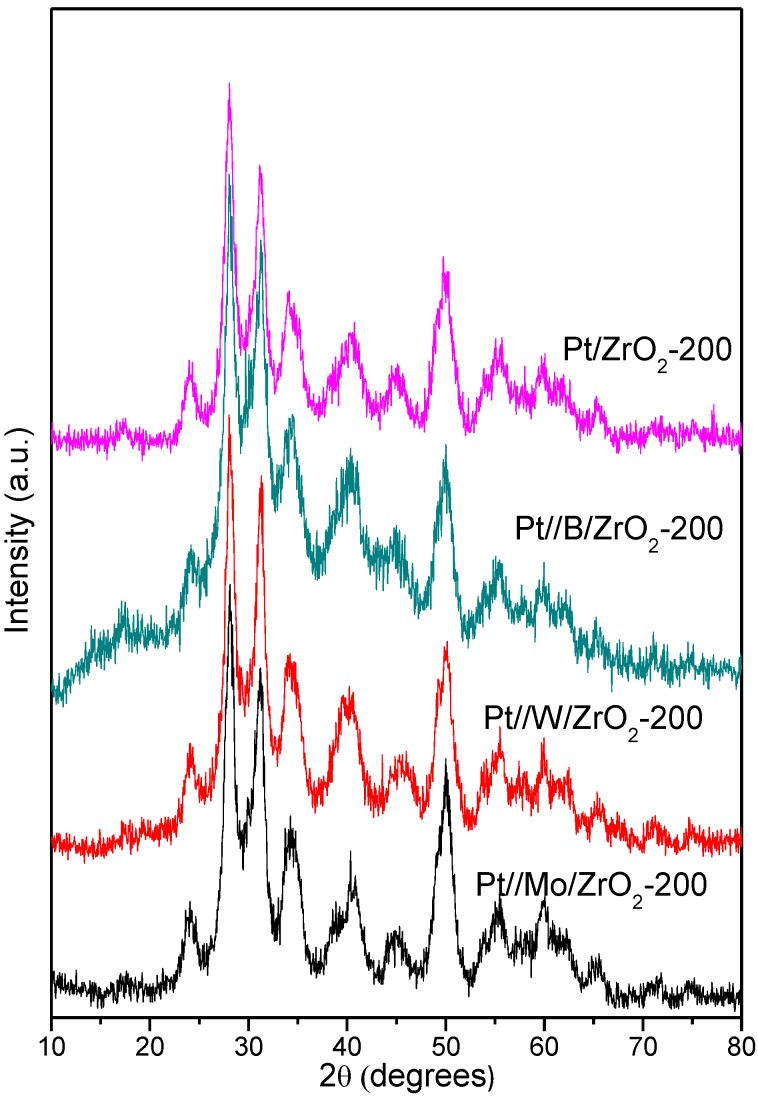
XRD patterns corresponding to catalysts used in this work.

Platinum signal at 2θ = 39.8° is partially overlapped with a ZrO_2_ band in XRD diffraction (Figure 1) and therefore it is somehow difficult to estimate the Pt particle size by applying the Scherrer equation. Instead, hydrogen chemisorption was used to estimate the Pt particle size, the results being shown in Table 1. The obtained particle size values were in the range between 4 nm for Pt/ZrO_2_ and 13 nm for Pt//Mo/ZrO_2_.

XRD patterns corresponding to Pt catalysts used in this work are presented in Figure 1. As can be observed, all catalysts present a minority tetragonal ZrO_2_ crystalline phase (PDF 14-0534) and a main monoclinic ZrO_2_ phase (baddeleyite, PDF 37-1484), while modifiers were not detected by this technique indicating that either modifiers are uniformly dispersed on the ZrO_2_ surface or the modifiers crystal size is smaller than detection limits of XRD (or a combination of both factors).

**Figure 2:**
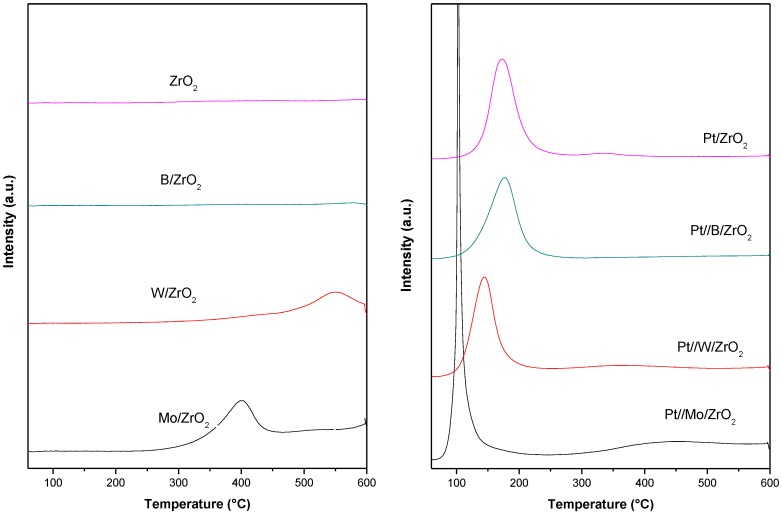
Temperature-programmed reduction (TPR) profiles for supports (**left**) and supported Pt catalysts (**right**).

TPR profiles of both supports and supported Pt catalysts are depicted in Figure 2. In the case of bare supports, Mo/ZrO_2_ presents a reduction peak at temperatures above 300 °C (centered at ca. 400 °C) while W/ZrO_2_ also shows a small reduction peak at temperatures even higher (ca. 550 °C). These high temperature bands can be attributed to the reduction of polyoxometalates structure [51]. As far as the supported Pt catalysts are concerned, all catalysts exhibited a low temperature reduction peak (below 200 °C) that can be associated to Pt^2+^ or Pt^4+^ reduction to Pt^0^ [53].

In the case of Pt//B/ZrO_2_, the reduction profile is quite similar to Pt/ZrO_2_ with a maximum over 175 °C. Therefore, the presence of B_2_O_3_ probably does not affect the reducibility of the metal particle. For the rest of the catalysts there are some changes in shape and/or reduction temperature of the Pt reduction species. Thus, the Pt//Mo/ZrO_2_ catalyst presents a very narrow reduction peak centered at 110 °C and a wide band at temperatures above 300 °C that could be associated to the Pt catalyzed reduction of Mo species. For Pt//W/ZrO_2_, the Pt reduction peak is similar in shape to that of Pt/ZrO_2_ but displaced to lower temperatures (apex at about 150 °C). Again, a small drift in the baseline at high temperatures is observed for this catalyst indicating some reduction/decomposition of W species.

Based on the above presented results, complete reduction of the incorporated platinum can be assumed after catalyst reduction at 200 °C, previous to its application in glycerol hydrogenolysis.

### 3.2. Surface Acid Properties of the Catalysts

**Figure 3:**
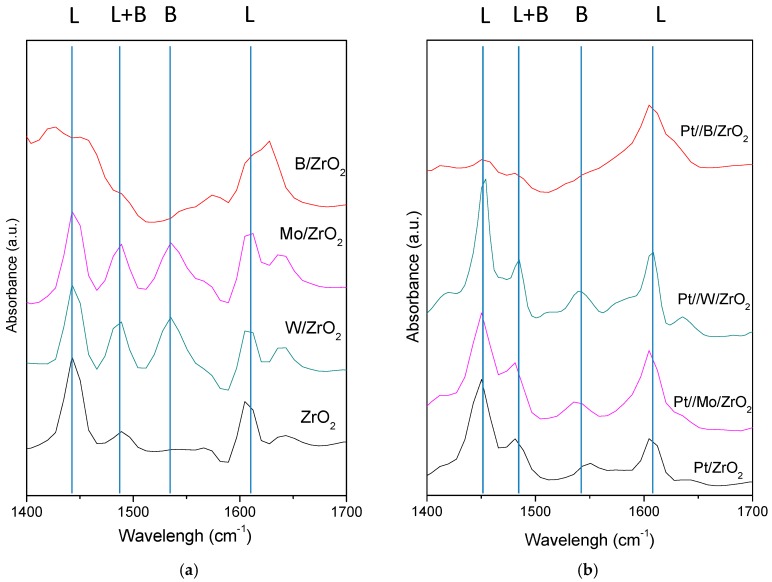
Diffuse reflection infrared spectroscopy of chemisorbed pyridine (Py-DRIFT) patterns obtained for supports (**a**) and supported Pt catalyst (**b**). Spectra were taken after pyridine adsorption and subsequent heating to 200 °C for 60 min.

The nature of the acid sites in calcined catalyst was analyzed by diffuse reflection infrared spectroscopy of chemisorbed pyridine (Py-DRIFT). Figure 3 presents the results obtained for each catalyst after pyridine adsorption and subsequent heating to 200 °C for 60 min.

As for the supports, Figure 3a shows that bare ZrO_2_ presents only Lewis acid sites while the incorporation of the modifiers induces the appearance of Brönsted acidity (band at 1542 cm^−1^) [17,54]. Borated zirconia is the exception since this solid could not absorb enough pyridine to obtain a clear pattern, consistent with the low acidity inferred from propan-2-ol test reaction detailed below.

On the other hand, as for supported Pt catalysts, the obtained Py-DRIFT profiles are presented in Figure 3b. These spectra show that Pt incorporation enhanced the Lewis acidity thus increasing the Lewis to Brönsted ratio [17]. In this line, Pt//Mo/ZrO_2_ presents a similar profile to Pt/ZrO_2_ while Pt//W/ZrO_2_ shows a more intense Lewis component than the above-mentioned catalysts. Finally, similarly to supports, the Pt//B/ZrO_2_ solid does not adsorb pyridine and therefore it has to be considered as a catalyst with very low acidity.

**Figure 4:**
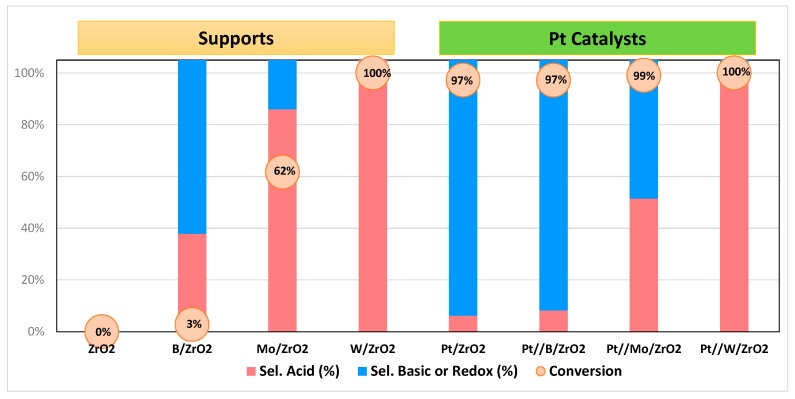
Propan-2-ol conversion and products distribution obtained in the gas-phase propan-2-ol transformation test reaction at 200 °C (time on stream, 2 h). Sel. Acid: selectivity to products formed on acid sites (propene and diisopropyl ether); Sel. Basic or Redox: selectivity to products formed on basic or redox sites (acetone and diacetone alcohol).

In addition, surface acid-base properties of both supports and Pt catalysts were determined through propan-2-ol decomposition test reaction (Figure 4 and Table S1 present the obtained results). This test reaction allows us to determine acid and basic properties of the catalysts by analyzing the products distribution formed upon propan-2-ol transformation. When the reaction takes place over acid sites, dehydration products (propene and/or diisopropyl ether) are produced, while if basic or redox sites are involved, acetone and/or diacetone alcohol (resulting from acetone self-condensation) are obtained [50].

The W/ZrO_2_ catalyst led to total conversion while B/ZrO_2_ and ZrO_2_ were nearly inactive (<5% and 0% respectively). Mo/ZrO_2_ exhibited an intermediate activity presenting around 60% conversion. As for the selectivity to the different reaction products, propene is the main product for all active (conversion over 5%) supports while Mo/ZrO_2_ is the only among those supports yielding acetone to some extent (18% selectivity, 62% conversion), probably due to the presence of some surface redox/basic sites.

Incorporation of Pt to the supports changes both the activity and selectivity of propan-2-ol transformation as compared to bare supports. Thus, the new metal active sites created upon Pt incorporation induced an increase in propan-2-ol conversion to values higher than 95% for all Pt catalysts, the selectivity depending on the catalyst support. More specifically, supported Pt over the less active supports (bare ZrO_2_ and B/ZrO_2_) yielded mainly acetone and diacetone alcohol due to the new redox (metallic) sites created in the solids and thus presenting a selectivity to propene or diisopropyl ether below 10%. An intermediate behavior was observed for Pt//Mo/ZrO_2_, yielding equally those products formed on acid sites (propene and diisopropyl ether) and those formed on basic/redox sites (mainly acetone). Finally, the Pt//W/ZrO_2_ catalyst exhibited a 100% propan-2-ol conversion with 100% selectivity to propene, thus confirming the acidic character already observed with the corresponding supports.

A comparison between data exposed in Figure 3 (Py-DRIFT) and Figure 4 (propan-2-ol transformation) indicates that those Pt catalysts whose supports presented enhanced Brönsted acidity (Mo/ZrO_2_ and W/ZrO_2_) lead to propan-2-ol dehydration to propene (or diisopropyl ether) while those catalysts whose supports showed a less marked Brönsted acidity (B/ZrO_2_ and ZrO_2_) exhibited high selectivity to acetone (or diacetone alcohol) due to the redox sites created during Pt incorporation.

To sum up, the acidity order of the solid was Pt//W/ZrO_2_ > Pt//Mo/ZrO_2_ >> Pt//B/ZrO_2_ = Pt/ZrO_2_.

### 3.3. Glycerol Conversion under Hydrogenolysis Conditions

**Figure 5:**
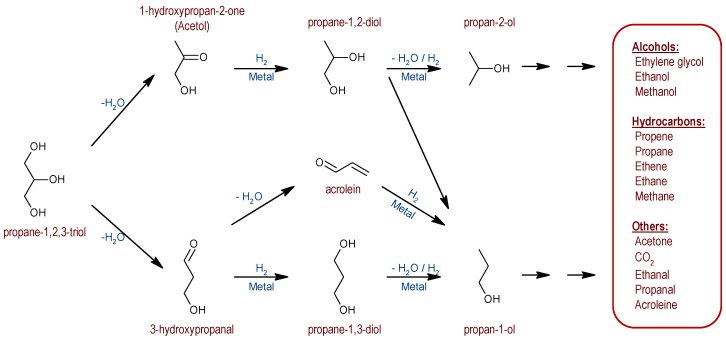
Glycerol hydrogenolysis pathways.

Liquid phase glycerol conversion under reductive atmosphere provides a wide range of products depending on the catalyst and the reaction conditions, such as reaction time, pressure or temperature. It has been reported in the literature that glycerol hydrogenolysis initiates with dehydration to either acetol (primary hydroxyl group dehydration) or 3-hydroxypropanal (secondary hydroxyl group dehydration) [13]. Then, subsequent hydrogenation of these carbonyl compounds leads to diols (1,2-propanediol or 1,3-propanediol respectively), and further dehydration/hydrogenation steps produce mono-alcohols (propan-1-ol or propan-2-ol). Secondary C-C breaking reactions are responsible for the formation of C2 and C1 products (Figure 5).

In order to evaluate the influence of the support on the catalytic activity on glycerol hydrogenolysis, a first screening of supported Pt catalysts was approached. Blank experiments without catalyst and with bare supports were carried out and, in all cases, the obtained glycerol conversion was less than 1% after 24 h of reaction. This means that, despite the acid nature of some supports as revealed by Py-DRIFT and propan-2-ol test reaction, metallic sites are necessary to promote glycerol dehydration, as already reported in the literature [24].

**Figure 6:**
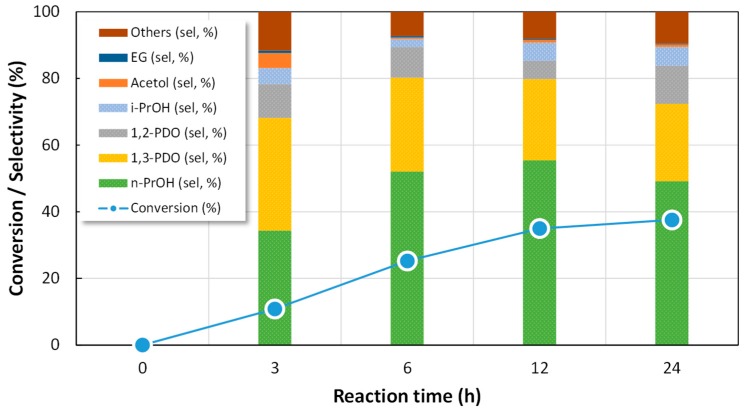
Reaction profile obtained in glycerol hydrogenolysis over Pt//W/ZrO_2_-200 at 180 °C, 6 bars of H_2_ initial pressure, 100 mg of catalyst and 10 mL of glycerol 1.36 M.

A time-dependent reaction profile was obtained for each Pt catalyst, by carrying out individual reactions at different reaction times (3, 6, 12 and 24 h). The obtained results were similar for all solids, Figure 6 presenting the profile obtained for Pt//W/ZrO_2_. Typically, glycerol conversion increases with reaction time up to 12 h of reaction and then the reaction rate slows down, so 12 h was selected as reaction time for further experiments. In terms of products distribution, as shown in Figure 6, only *n*-propanol production steadily increases with the reaction time, while the other reaction products were formed at the beginning and then hardly changed with reaction time. Thus, apparently, longer reaction time will provide high yield to mono-alcohols while shorter reaction time would increase the selectivity to diols.

**Figure 7:**
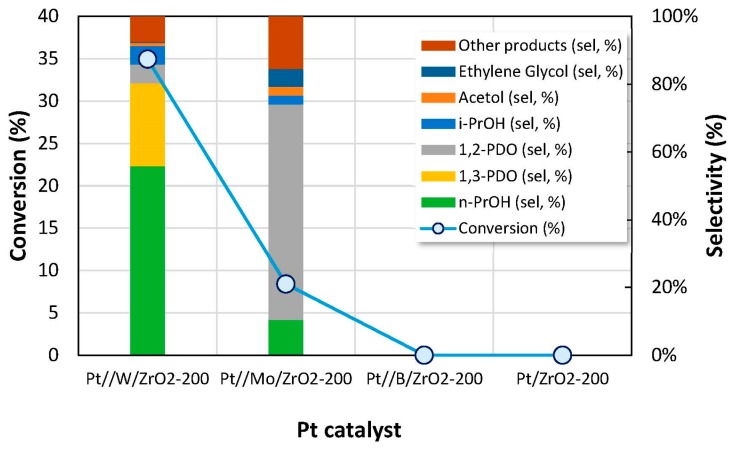
Glycerol conversion and products selectivity obtained for the synthesized catalysts after 12 h of reaction in the liquid-phase glycerol hydrogenolysis at 180 °C. Six bar of H_2_ initial pressure, 100 mg of catalyst and 10 mL of glycerol 1.36 M.

Figure 7 shows the results obtained for all the supported Pt catalysts prepared in this work when essayed in the liquid-phase glycerol hydrogenolysis (reaction time, 12 h). Regarding catalytic conversion, the most active catalyst was Pt//W/ZrO_2_ (35% conv.) followed by Pt//Mo/ZrO_2_ (7% conv). It is important to note that, as shown in Figure 4, in the propan-2-ol transformation test reaction, Pt//W/ZrO_2_ and Pt//Mo/ZrO_2_ were the only Pt catalysts yielding propene and/or diethyl ether, that is, products formed over surface acid sites. Among them, Pt//Mo/ZrO_2_ also yielded acetone (45% yield), product formed on basic/redox sites.

On the other hand, Pt//B/ZrO_2_ and Pt/ZrO_2_ catalysts were inactive in glycerol hydrogenolysis under the described reaction conditions (Figure 7). These catalysts mainly yielded acetone and/or diacetone alcohol (products formed over basic/redox sites) in propan-2-ol test reaction. Thus, it is hardly surprising that both catalysts were inactive in glycerol dehydration to acetol, since no activity in propan-2-ol dehydration to propene was reported (Figure 4).

Therefore, although the propan-2-ol transformation is carried out in the gas phase, and glycerol hydrogenolysis under hydrothermal reaction conditions, there is a good correlation between both reactions, that is, the results obtained from propan-2-ol transformation test reaction could provide some relevant information for the more complex glycerol hydrogenolysis. Thus, considering the above exposed results, those Pt catalysts exhibiting high selectivity to acid-derived products in propan-2-ol test reaction are expected to be active in glycerol hydrogenolysis. However, this correlation does not seem to work for the bare supports, since, despite the fact that W/ZrO_2_ and Mo/ZrO_2_ supports were active in the propan-2-ol dehydration, they were not active in glycerol hydrogenolysis. This confirms that the metal function is critical for the catalyst to be active in glycerol hydrogenolysis. In this sense, as presented above, the incorporation of Pt into the support induces the creation of a new type of Lewis acid sites, associated or in close contact to Pt particles. In a previous study, our research group examined the role and origin of catalyst acidity and observed that chlorinated precursors were responsible for the catalysts acidity and, when non-chlorinated precursors were used, acidity observed was negligible [17]. It has to be assumed that these Lewis acid sites in close contact or related with Pt metal sites are responsible for glycerol conversion under reductive conditions.

As for the selectivity to different reaction products in glycerol conversion, Figure 7 shows that Pt//W/ZrO_2_ was the only catalyst leading to 1,3-PDO (24% sel.) although its main reaction product was *n*-propanol (55% sel.). It has been reported that 1,3-PDO is usually obtained in the presence of specific metallic compounds, among them W is the most common, the reaction taking place either via glycerol secondary hydroxyl group dehydration and subsequent hydrogenation (Figure 5) or, alternatively, by a direct hydrogenolysis mechanism [12,13]. On the other hand, the main reaction product obtained in the reaction using the Pt//Mo/ZrO_2_ catalyst was 1,2-PDO with 49% selectivity. It is worth noting that, for all the active catalysts, the selectivity to acetol was less than 2% and *i*-propanol was also obtained with selectivity ranging 2%–13%. Different products such as methanol, ethanol or acetic acid were also formed and included in ‘other products’ selectivity. Finally, additional reaction products were detected in the gas-phase; carbon dioxide and C1-C3 hydrocarbons (mainly propane and propene) were identified in the gas phase, probably produced via glycerol aqueous phase reforming (APR) under hydrothermal reaction conditions [55].

#### 3.3.1. Influence of the Reaction Temperature

The liquid-phase glycerol conversion was essayed at 160, 180 and 200 °C in order to study the influence of the reaction temperature on catalytic activity and products distribution over the supported Pt catalysts synthesized in this work, the results being presented in Table 2.

These results indicate that Pt//W/ZrO_2_ is the most active catalyst at any temperature, being the only active catalyst at 160 °C with ca. 20% conversion. At this temperature, the major reaction product is 1,3-PDO with 42% selectivity. According to the results reported in the literature, for tungsten-based catalysts, 1,3-PDO production is the consequence of an interaction between the noble metal (Pt in our case) and W, following a direct hydrogenolysis reaction mechanism [12,13]. For Pt//W/ZrO_2_, these interactions are evidenced in TPR experiments by the shift to a lower reduction temperature of the W reduction band (Figure 2).

At 180 °C, Pt//W/ZrO_2_ is again the most active catalyst in terms of conversion (ca. 35%), while Pt//Mo/ZrO_2_ exhibits 7% glycerol conversion. On the other hand, Pt//B/ZrO_2_ and Pt/ZrO_2_ catalysts are still inactive. In terms of selectivity to diols at 180 °C, Pt//W/ZrO_2_ also yields 1,3-PDO (23% sel.) but, at this temperature, the main reaction product is *n*-propanol (52% sel.), indicative of a deeper hydrogenolysis process [56]. Pt//Mo/ZrO_2_ is the best option for 1,2-PDO production at 180 °C with 49% selectivity.

Finally, at 200 °C all tested catalysts are active in glycerol hydrogenolysis, conversions ranging from 5% to 54%. The catalytic activity obtained, after 12 h of reaction, follows the order:

Pt//W/ZrO_2_ (54% conv.) > Pt//Mo/ZrO_2_ (8%) = Pt//B/ZrO_2_ (8%) > Pt/ZrO_2_ (5%).

As for products distribution, at 200 °C there is an increase in the selectivity to the so-called ‘other products’ or even to ‘gas phase products’, pointing towards diols consumption at higher temperatures as a consequence of the undesired APR processes [53,54]. Again, Pt//W/ZrO_2_ is the only catalyst yielding 1,3-PDO but with worse selectivity (12% sel.) than that obtained at lower temperatures. As mentioned above, C-3 hydrocarbons (propane and propene) were the main components in the gas-phase fraction, its selectivity increasing with the reaction temperature.

#### 3.3.2. Acetol Conversion under Reaction Conditions

**Figure 8:**
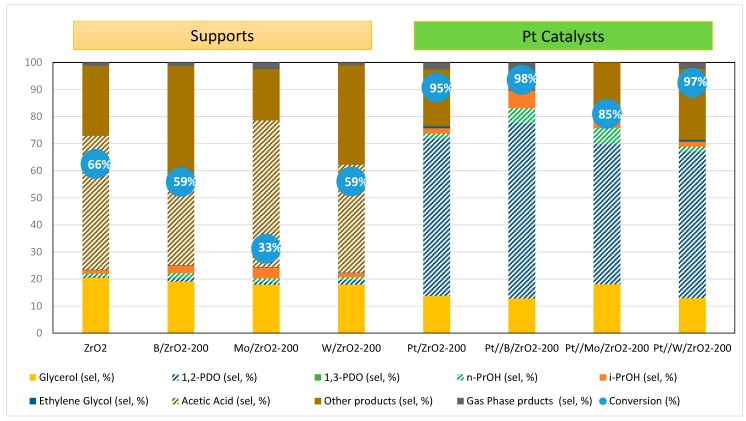
Acetol conversion and products selectivity obtained for bare supports and reduced Pt catalysts in the liquid-phase acetol transformation under reductive atmosphere. Reaction conditions: catalyst/support, 100 mg; H_2_ initial pressure, 6 bars; reaction temperature, 180 °C; reaction time, 4 h; aqueous acetol 0.5 M (10 mL).

In order to gather additional information on the origin of products distribution in glycerol hydrogenolysis, some additional reactions with acetol as the starting reagent were carried out, the results being presented in Figure 8 for both supports and supported Pt catalysts.

High acetol conversion was obtained for all supports, ranging from 33% for Mo/ZrO_2_ to 66% conversion for ZrO_2_. In all cases, the modification of the ZrO_2_ with acidic additives leads to a fall in catalytic activity while selectivity patterns are quite similar. For all supports, selectivity to 1,2-PDO was less than 2% at the best, the main reaction product being acetic acid (30%–54% sel.). Moreover, acetol rehydration was also observed, with selectivity to glycerol around 20%. As stated above, in spite of the enhanced acidic properties above presented, synthesized supports (without metal phase) cannot convert glycerol, although they do convert acetol but with selectivity to diols lower than 2%.

Moreover, incorporation of Pt metal boosted acetol conversion with a drastic change in the products distribution profile, 1,2-PDO being the main reaction product formed at selected reaction conditions. As expected, among the reaction products 1,3-PDO is not obtained even for the Pt//W/ZrO_2_ catalyst. It seems to be clear that some kind of metal-support interaction plays an important role in the products distribution profile (selectivity) of the process [57,58], and it is likely that this interaction involves the acid sites of the support in close contact with Pt particles. In this line, Du et al. [59] demonstrated that this interaction is responsible for an activity enhancement through a synergistic effect. In our case, attending to the acidic properties described above, it seems that Brönsted acidity created after modifiers incorporation is responsible for the acetol conversion to non-diols products while, on the other hand, Lewis acid sites created after Pt incorporation enhances both glycerol and acetol conversion to 1,2-PDO. Finally, there were some other reaction products formed, including methanol, ethanol or condensation products like furan derivatives obtained by retro-aldol dimerization of acetol [58].

In parallel to the above commented reactions, additional experiments with 1,2-PDO as starting substrate were carried under the same reaction conditions. These experiments did not show any catalytic activity, so further experiments were performed using higher 1,2-PDO concentration and longer reaction time (1 M solution and 12 h). It was observed that, even in the presence of Pt metal, 1,2-PDO is a stable product except for Pt//W/ZrO_2_ catalyst that converts approximately 33% of the diol into *n*-propanol and other gaseous products (mainly C-3 hydrocarbons). It is relevant to state that mono-alcohols (*n*-PrOH and *i*-PrOH) were present for all catalysts in glycerol or acetol reactions but, on the contrary, they were not observed when 1,2-PDO is used as starting substrate, except for Pt//W/ZrO_2_. Among all modifiers studied in this work, the W-based one clearly breaks the trend in products selectivity. According to the bibliography, the oxophilicity of W enhances the direct hydrogenolysis mechanism through a triple-action: (1) it acts as a strong anchoring site for the terminal OH group/s of glycerol forming a terminal alkoxide, (2) it provides the necessary Brönsted acid sites to protonate the internal OH group of glycerol, and (3) it stabilizes the secondary carbocation formed, avoiding the degradation of the 1,3-PDO product [12,60]. Nevertheless, although the Mo based system could also be susceptible to performing this triple effect, results observed are far from those obtained for W. In this sense, Liao et al. [61] described that the presence of Mo in the Ru catalyst can reduce the metal function activity. Assuming that something similar may occur with our catalyst, the dehydration-hydrogenation mechanism would be favored instead of direct hydrogenolysis, thus leading to 1,2-PDO as the main reaction product.

The differential behavior of the Pt//W/ZrO_2_ catalyst led us to design additional experiments in order to gain knowledge on the stability of this catalyst under reaction conditions.

#### 3.3.3. Reusability Study for Pt//W/ZrO_2_

**Figure 9:**
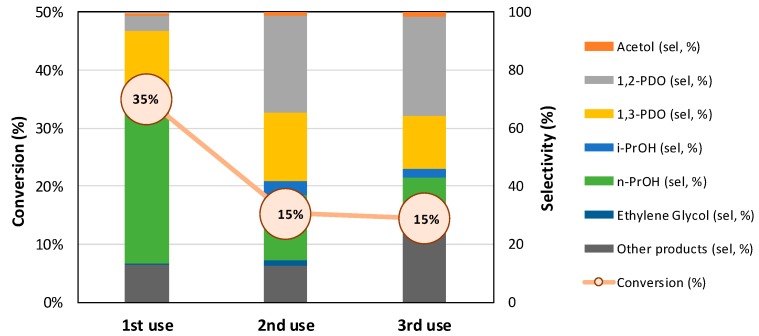
Reusability tests for the Pt//W/ZrO_2_ catalyst in the liquid-phase glycerol hydrogenolysis under the following reaction conditions: reaction temperature, 180 °C; H_2_ initial pressure, 6 bars; reaction time, 24 h; catalyst, 100 mg; aqueous glycerol 1.36 M (10 mL).

In order to perform reusability tests, spent Pt//W/ZrO_2_ catalyst was recovered by vacuum filtration, washed with 3 × 25 mL of ultrapure water, dried overnight at 110 °C and reduced at 200 °C before being reused in glycerol hydrogenolysis. As can be observed in Figure 9, hydrothermal reaction conditions have a detrimental effect on the catalyst performance with a drop-in glycerol conversion from 35% in the first run to 15% conversion in the second one. Moreover, an important effect on products selectivity is revealed and thus fresh catalyst yielded *n*-PrOH as main product (52% sel.) followed by 1,3-PDO (23% sel.) and 1,2-PDO (5% sel). However, for the spent catalyst (second use), 1,2-PDO was the main reaction product (33% sel.) while 1,3-PDO selectivity remained at 23% and *n*-PrOH fell down to 22%. Finally, during the third use, catalytic activity remains stable at 15% glycerol conversion. As for products distribution, while the selectivity to 1,2-PDO remains constant (34% sel.), the selectivity to 1,3-PDO has a slight decrease, down to 18%. In this third use, the *n*-PrOH selectivity fell again to 13% while selectivity to other products steadily increases from the first to the third reaction run.

**Figure 10:**
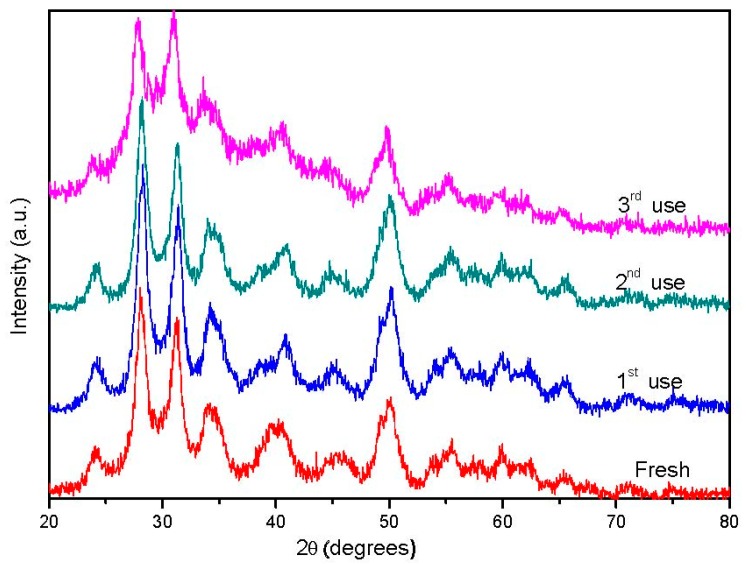
XRD patterns obtained for the Pt//W/ZrO_2_ catalyst in the reusability test.

**Figure 11:**
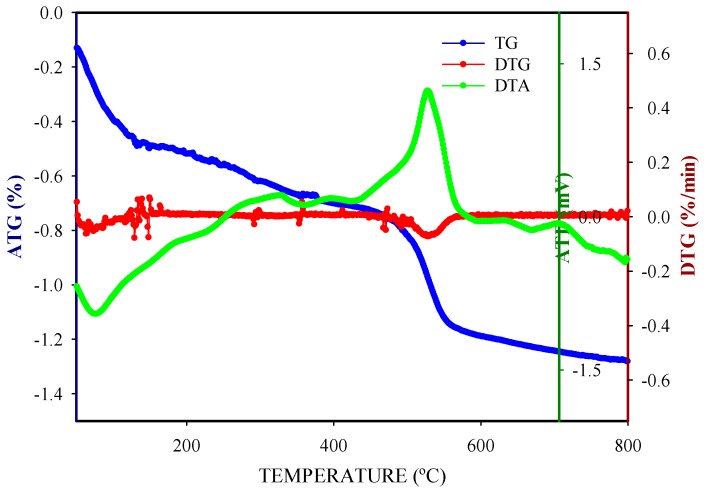
TGA-DTA-DTG analysis for spent Pt//W/ZrO_2_ catalyst.

The observed fall in catalytic activity during catalyst reutilization could be associated to either deactivation caused by carbonaceous deposits or to the lixiviation of W or Pt species from the catalyst. XRD patterns obtained for fresh and reused catalysts did not show any significant difference, except for an attenuation of the ZrO_2_ bands after the third reaction run, that could be associated to coke fouling or to crystalline phase degradation under hydrothermal reaction conditions (Figure 10). In this line, ATG-DTA-DTG experiments carried out for spent Pt//W/ZrO_2_ catalyst (Figure 11) exhibited a high temperature exothermic signal that strengthens the hypothesis of carbonaceous residues deposited on the catalyst surface. Nevertheless, the limited weight loss observed along the experiment (overall mass lost ca. 1.2%) indicates that the fouling process is not the main cause of deactivation.

ICP-MS analysis for the filtered reaction liquid-phase as well as for the recovered catalyst after the first use was also carried out (Table 3). The obtained results revealed the presence of W species in the aqueous phase (0.80%), thus proving that some lixiviation of the W species occurs during the first reaction. On the contrary, less than 0.01% Pt was observed in the liquid phase. Therefore, it has to be assumed that W lixiviation would be the main cause for the loss of both glycerol conversion and selectivity to 1,3-PDO in the Pt//W/ZrO_2_ catalyst after its first use. Finally, in order to rule out homogeneous catalysis by lixiviated W species, an additional experiment was performed with the equivalent amount of the W precursor dissolved in the reaction media, obtaining negligible glycerol conversion, thus indicating of the absence of homogeneous catalysis.

## 4. Conclusions

Sol-gel synthesized ZrO_2_ was modified by the incorporation of different additives in order to obtain supports with enhanced acidic properties for subsequent Pt impregnation (5% *w*/*w*, nominal). In this way, Pt/ZrO_2_, Pt//B/ZrO_2_, Pt//W/ZrO_2_ and Pt//Mo/ZrO_2_ catalysts were synthesized, characterized and tested for glycerol hydrogenolysis.

Modifiers incorporation increased Brönsted acidity of ZrO_2_ (except for B/ZrO_2_) while further incorporation of Pt improved Lewis acidity. Glycerol hydrogenolysis showed a correlation between glycerol conversion and catalysts acidity, as obtained from the propan-2-ol test reaction.

In glycerol hydrogenolysis, Pt//W/ZrO_2_ was the most active catalyst followed by Pt//Mo/ZrO_2_ while Pt/ZrO_2_ and Pt//B/ZrO_2_ were nearly inactive, in agreement with their low acidity. As for the selectivity, the major reaction product obtained depends on the modifier. Thus, Pt//W/ZrO_2_ is the only catalyst able to produce 1,3-PDO although *n*-propanol is the main reaction product. On the other hand, Pt//Mo/ZrO_2_ led to 1,2-PDO mainly, while Pt//B/ZrO_2_ gave high selectivity to gas-phase products, mainly C-3 hydrocarbons.

Reactions with acetol or 1,2-PDO as starting substrates provided some additional information on the reaction mechanism. Thus, both supports and supported Pt catalysts were found to be active in acetol hydrogenation with opposite behavior. Supports mainly yielded acetic acid and oligomerization products while supported Pt catalysts led to 1,2-PDO as result of acetol hydrogenation. Finally, 1,2-PDO was found to be stable under reaction conditions, Pt//W/ZrO_2_ being the only catalyst able to induce 1,2-PDO hydrogenolysis yielding *n*-propanol and gas-phase products (C-3 hydrocarbons).

Regarding the reusability study carried out with Pt//W/ZrO_2_, glycerol conversion dropped in the first reuse, staying constant for the second one. As for the selectivity, successive reuses led to an increase in 1,2-PDO associated to a decrease of *n*-PrOH, selectivity to 1,3-PDO remaining almost constant. Changes in activity could be explained by some lixiviation of W species as detected by ICP-MS analysis of the resulting liquid-phase or it could be caused by carbonaceous deposits on the catalyst evidenced by TGA-TDA.

## Figures and Tables

**Table 1:** Some features concerning the characterization of the different supported Pt catalysts. Brunnauer–Emmett–Teller (BET) surface area, Pt particle size (H_2_ chemisorption) and catalyst chemical composition (as determined by X-ray photoelectron spectroscopy (XPS) and inductively coupled plasma with mass spectrometry detection (ICP-MS)).

Catalyst	S_BET_ (m^2^·g^−1^)	Metal ^a^ Content (at.%) ICP-MS	Pt Content (wt.%) ICP-MS	Pt Particle Size (nm) H_2_ Chemisorp.	XPS (at.%)				
					Zr (%)	M ^a^ (%)	Cl (%)	O (%)	Pt (%)
**Pt/ZrO_2_**	109	-	4.8	4	27.76	-	11.74	59.26	1.24
**Pt//B/ZrO_2_**	93	11	4.6	7	25.61	7.70	11.74	53.85	1.10
**Pt//Mo/ZrO_2_**	86	7.0	4.6	13	28.11	2.31	11.37	57.45	0.76
**Pt//W/ZrO_2_**	86	8.3	4.6	8	27.42	1.55	10.00	59.96	1.07

^a^ metal = (B, Mo or W).

**Table 2:** Liquid-phase hydrogenolysis of glycerol at different temperatures. Conversion and selectivity to propan-2-ol (*i*-PrOH), propan-1-ol (*n*-PrOH), acetol, propane-1,2-diol (1,2-PDO), propane-1,3-diol (1,3-PDO), ethylene glycol (EG) and other reaction products.

Catalyst	Temp.	Conv.	Products Selectivity (%)							
			*i*-PrOH	*n*-PrOH	Acetol	1,2-PDO	1,3-PDO	EG	Others	Gas Phase
Pt//Mo/ZrO_2_-200	200	8.4	0.0	5.1	7.6	6.6	0.0	0.9	60.5	19.3
	180	6.7	2.3	8.1	2.0	48.9	0.0	4.3	13.3	21.2
	160	0.0	0.0	0.0	0.0	0.0	0.0	0.0	0.0	0.0
Pt//W/ZrO_2_-200	200	53.8	8.0	40.5	1.2	7.2	12.3	0.3	14.7	15.8
	180	34.5	5.1	52.4	0.8	5.2	23.0	0.3	7.1	6.1
	160	19.7	2.9	34.5	0.3	4.2	42.1	0.1	6.4	9.5
Pt//B/ZrO_2_-200	200	8.3	3.8	12.2	5.9	14.0	0.0	11.5	23.3	29.2
	180	0.0	0.0	0.0	0.0	0.0	0.0	0.0	0.0	0.0
	160	0.0	0.0	0.0	0.0	0.0	0.0	0.0	0.0	0.0
Pt/ZrO_2_-200	200	5.4	3.8	10.7	8.9	18.9	0.0	18.0	39.8	>1
	180	0.0	0.0	0.0	0.0	0.0	0.0	0.0	0.0	0.0
	160	0.0	0.0	0.0	0.0	0.0	0.0	0.0	0.0	0.0

Reaction Conditions: 100 mg of Pt Catalyst; 6 bar of initial hydrogen pressure; reaction time, 12 h; Glycerol concentration 1.36 M (aqueous, 10 mL).

**Table 3:** ICP-MS data obtained during the reusability test for Pt//W/ZrO_2_-200 in glycerol hydrogenolysis. Comparison between Pt and W content (weight, %) in fresh and first run recovered catalyst (used) and metal lixiviated to the reaction aqueous phase.

	Pt (wt.%)	W (wt.%)
Pt//W/ZrO_2_-Fresh	4.60	8.32
Pt//W/ZrO_2_-Used	4.58	7.46
Aqueous Phase	0.01	0.80

## Supplementary Materials

The following are available online at https://www.mdpi.com/2079-4991/9/4/509/s1, Table S1: Propan-2-ol conversion and products distribution obtained in the gas-phase propan-2-ol transformation test reaction at 200 °C (time on stream, 2 h). Sel. Acid: selectivity to products formed on acid sites (propene and diisopropyl ether); Sel. Basic or Redox: selectivity to products formed on basic or redox sites (acetone and diacetone alcohol).

## References

[1] Russbueldt B.M.E., Hoelderich W.F. (2010). New rare earth oxide catalyst for the transesterification of triglycerides with methanol resulting in biodediesel and pure glycerol. J. Catal..

[2] Besson M., Gallezot P., Pinel C. (2014). Conversion of biomass into chemicals over metal catalysts. Chem. Rev..

[3] Talebian-Kiakalaieh A., Amin N.A.S., Hezaveh H. (2014). Glycerol for renewable acrolein production by catalytic dehydration. Renew. Sustain. Energy Rev..

[4] Werpy T., Petersen G. (2004). Top Value Added Chemicals from Biomass Volume I—Results of Screening for Potential Candidates from Sugars and Synthesis Gas.

[5] Pagliaro M., Rossi M. (2008). The Future of Glycerol: New Uses of a Versatile Raw Material.

[6] Johnson D.T., Taconi K.A. (2007). The glycerin glut: Options for the value-added conversion of crude glycerol resulting from biodiesel production. Environ. Prog..

[7] Gholami Z., Abdullah A.Z., Lee K.T. (2014). Catalytic upgrading to polyglycerols and other value-added products. Renew. Sustain. Energy Rev..

[8] Chaminand J., Djakovitch L.A., Gallezot P., Marion P., Pinel C., Rosier C. (2004). Glycerol hydrogenolysis on heterogeneous catalysts. Green Chem..

[9] Vila F., Granados M.L., Ojeda M., Fierro J.L.G., Mariscal R. (2012). Glycerol hydrogenolysis to 1,2-propanediol with Cu/g-Al2O3: Effect of the activation process. Catal. Today.

[10] Quispe C.A.G., Coronado C.J.R., Carvalho J. (2013). Glycerol: Production, consumption, prices, characterization and new trends in combustion. Renew. Sustain. Energy Rev..

[11] Pagliaro M., Ciriminna R., Kimura H., Rossi M., Pina C.D. (2009). Recent advances in the conversion of bioglycerol into value-added products. Eur. J. Lipid Sci. Technol..

[12] Nakagawa Y., Shinmi Y., Koso S., Tomishige K. (2010). Direct Hydrogenolysis of Glycerol into 1,3-Propanediol over Rhenium-modified Iridium Catalyst. J. Catal..

[13] Feng J., Xu B. (2014). Reaction mechanism for the hetrogeneous hydrogenolysis of biomass-derived glicerol to propanediols. Prog. React. Kinet. Mech..

[14] Vasiliadou E.S., Lemonidou A.A. (2014). Glycerol transformation to value added C3 diols: Reaction mechanism, kinetic, and engineering aspects. WIREs Energy Environ..

[15] Yuan Z., Wu P., Gao J., Lu X., Hou Z., Zheng X. (2009). Pt7solid-base: A predominant catalyst for glycerol hydrogenolysis in a base-free aqueous solution. Catal. Lett..

[16] Marinas A., Bruijnincx P., Ftouni J., Urbano F.J., Pinel C. (2015). Sustainability metrics for a fossil- and renewable-based route for 1,2-propanediol production: A comparison. Catal. Today.

[17] Montes V., Checa M., Marinas A., Boutonnet M., Marinas J.M., Urbano F.J., Järas S., Pinel C. (2014). Synthesis of different ZnO-supported metal systems through microemulsion technique and application to catalytic transformation of glycerol to acetol and 1,2-propanedio. Catal. Today.

[18] Zhu S., Zhu Y., Hao S., Chen L., Zhang B., Li Y. (2012). Aqueous-phase hydrogenolysis of glycerol to 1,3-propanediol over Pt-H_4_SiW_12_O_40_/SiO_2_. Catal. Lett..

[19] Priya S., Kumar V., Kantam M., Bhargava S., Chary K. (2014). Vapour-phase hydrogenolysis of glycerol to 1,3-propanediol over supported pt catalysts: The effect of supports on the catalytic functionalities. Catal. Lett..

[20] Nakagawa Y., Tomishige K. (2011). Catalyst development for the hydrogenolysis of biomass derived chemicals to value-added ones. Catal. Surv. Asia.

[21] Montes V., Boutonnet M., Järås S., Lualdi M., Marinas A., Marinas J.M., Urbano F.J., Mora M. (2014). Preparation and characterization of Pt-modified Co-based catalysts through the microemulsion technique: Preliminary results on the Fischer-Tropsch synthesis. Catal. Today.

[22] Nakagawa Y., Tamura M., Tomishige K. (2014). Catalytic materials for the hydrogenolysis of glycerol to 1,3-propanediol. J. Mater. Chem. A.

[23] Checa M., Auneau F., Hidalgo-Carrillo J., Marinas A., Marinas J.M., Pinel C., Urbano F.J. (2012). Catalytic transformation of glycerol on several metal systems supported on ZnO. Catal. Today.

[24] Checa M., Marinas A., Marinas J.M., Urbano F.J. (2015). Deactivation study of supported Pt catalyst on glycerol Hydrogenolysis. Appl. Catal. A Gen..

[25] Dam J., Djanashvili K., Kapteijn F., Hanefeld U. (2013). Pt/Al_2_O_3_ Catalyzed 1,3-Propanediol Formation from Glycerol using Tungsten Additives. ChemCatChem.

[26] Hu J., Liu X., Wang B., Pei Y., Qiao M., Fan K. (2012). Reforming and Hydrogenolysis of Glycerol over Ni/ZnO Catalysts Prepared by Different Methods. Chin. J. Catal..

[27] Mallesham B., Sudarsanam P., Reddy B.V.S., Reddy B.M. (2016). Development of cerium promoted copper–magnesium catalysts for biomass valorization: Selective hydrogenolysis of bioglycerol. Appl. Catal. B Environ..

[28] Li Y., Liu H., Ma L., He D. (2014). Glycerol hydrogenolysis to propanediols over supported Pd–Re catalysts. RSC Adv..

[29] Zhu S., Gao X., Zhu Y., Li Y. (2015). Promoting effect of WOx on selective hydrogenolysis of glycerol to 1,3-propanediol over bifunctional Pt–WOx/Al_2_O_3_ catalysts. J. Mol. Catal. A Chem..

[30] Kittisakmontree P., Yoshida H., Fujita S., Arai M., Panpranot J. (2015). The effect of TiO_2_ particle size on the characteristics of Au-Pd/TiO 2 catalysts. Catal. Commun..

[31] Zanin C.I.C.B., Jordao E., Mandelli D., Figueiredo F.C.A., Carvalho W.A., Oliveira E.V. (2015). Hydrogenolysis of glycerol to alcohols catalyzed by transition metals supported on pillared clay. React. Kinet. Mech. Catal..

[32] García-Fernández S., Gandarias I., Requies J., Güemez M.B., Bennici S., Auroux A., Arias P.L. (2015). New approaches to the Pt/WO_x_/Al_2_O_3_ catalytic system behavior for the selective glycerol hydrogenolysis to 1,3-propanediol. J. Catal..

[33] Zhou W., Zhao Y., Wang Y., Wang S., Ma X. (2016). Glycerol Hydrogenolysis to 1,3-Propanediol on Tungstate/Zirconia-Supported Platinum: Hydrogen Spillover Facilitated by Pt(1 1 1) Formation. ChemCatChem.

[34] Ciftci A., Peng B., Jentys A., Lercher J.A., Hensen E.J.M. (2012). Support effects in the aqueous phase reforming of glycerol over supported platinum catalysts. Appl. Catal. A Gen..

[35] Choi Y., Park H., Yun Y.S., Yi J. (2015). Effects of Catalyst Pore Structure and Acid Properties on the Dehydration of Glycerol. ChemSusChem.

[36] Dam J.T., Kapteijn F., Djanashvili K., Hanefeld U. (2011). Tuning selectivity of Pt/CaCO_3_ in glycerol hydrogenolysis-A Design of Experiments approach. Catal. Commun..

[37] Montassier C., Dumas J.M., Granger P., Barbie J. (1995). Deactivation of supported copper based catalysts during polyol conversion in aqueous phase. Appl. Catal. A Gen..

[38] Helwani Z., Othman M.R., Aziz N., Kim J., Fernando W.J.N. (2009). Solid heterogeneous catalysts for transesterification of triglycerides with methanol: A review. Appl. Catal. A Gen..

[39] Osatiashtiani A., Lee A.F., Brown D.R., Melero J.A., Morales G., Wilson K. (2014). Bifunctional SO4/ZrO_2_ catalysts for 5-hydroxymethylfufural (5-HMF) production from glucose. Catal. Sci. Technol..

[40] Sinhamahapatra A., Pal P., Tarafdar A., Bajaj H.C., Panda A.B. (2013). Mesoporous Borated Zirconia: A Solid Acid-Base Bifunctional Catalyst. ChemCatChem.

[41] Pizzio L., Vázquez P., Cáceres C., Blanco M. (2001). Tungstophosphoric and molybdophosphoric acids supported on zirconia as esterification catalysts. Catal. Lett..

[42] Zhou W., Luo J., Wang Y., Liu J., Zhao Y., Wang S., Ma X. (2019). WO_x_ domain size, acid properties and mechanistic aspects of glycerol hydrogenolysis over Pt/WO_x_/ZrO_2_. Appl. Catal. B.

[43] Fan Y., Cheng S., Wang H., Tian J., Xie S., Pei Y., Qiao M., Zong B. (2017). Pt–WOx on monoclinic or tetrahedral ZrO2: Crystal phase effect of zirconia on glycerol hydrogenolysis to 1,3-propanediol. Appl. Catal. B.

[44] Zhu S., Gao X., Zhu Y., Zhu Y., Xiang X., Hu C., Li Y. (2013). Alkaline metals modified Pt–H_4_SiW_12_O_40_/ZrO_2_ catalysts for the selective hydrogenolysis of glycerol to 1,3-propanediol. Appl. Catal. B.

[45] Zhu S., Zhu Y., Hao S., Zheng H., Mo T., Li Y. (2012). One-step hydrogenolysis of glycerol to biopropanols over Pt-H_4_SiW_12_O_40_/ZrO_2_ catalysts. Green Chem..

[46] Gong L., Lu Y., Ding Y., Lin R., Li J., Dong W., Wang T., Chen W. (2010). Selective hydrogenolysis of glycerol to 1,3-propanediol over a Pt/WO_3_/TiO_2_/SiO_2_ catalyst in aqueous media. Appl. Catal. A Gen..

[47] Huang L., Zhu Y., Zheng H., Ding G., Li Y. (2009). Direct conversion of glycerol into 1,3-propanediol over Cu-H_4_SiW_12_O_40_/SiO_2_ in vapor phase. Catal. Lett..

[48] Arundhathi R., Mizugaki T., Mitsudome T., Jitsukawa K., Kaneda K. (2013). Highly selective hydrogenolysis of glycerol to 1,3-propanediol over a boehmite-supported platinum/tungsten catalyst. ChemSusChem.

[49] Zhu S., Qiu Y., Zhu Y., Hao S., Zheng H., Li Y. (2013). Hydrogenolysis of glycerol to 1,3-propanediol over bifunctional catalysts containing Pt and heteropolyacids. Catal. Today.

[50] Aramendia M.A., Borau V., Jimenez C., Marinas J.M., Porras A., Urbano F.J. (1997). Synthesis and characterization of ZrO_2_ as an acid-base catalyst: Dehydration-dehydrogenation of propan-2-ol. J. Chem. Soc. Faraday Trans..

[51] Haffad D., Chambellan A., Lavalley J.C. (2001). Propan-2-ol transformation on simple metal oxides TiO_2_, ZrO_2_ and CeO_2_. J. Mol. Catal. A Chem..

[52] Alves-Rosa M.A., Martins L., Hammer P., Pulcinelli S.H., Santilli C.V. (2014). Structure and catalytic properties of sulfated zirconia foams. J. Sol-Gel Sci. Technol..

[53] Consonni M., Jokic D., Murzin D.Y., Touroude R. (1999). High performances of Pt/ZnO catalysts in selective hydrogenation of crotonaldehyde. J. Catal..

[54] Miranda M., Ramírez S.A., Jurado S.G., Vera C.R. (2015). Superficial effects and catalytic activity of ZrO_2_–SO_4_^2−^ as a function of the crystal structure. J. Mol. Catal. A Chem..

[55] Seretis A., Tsiakaras P. (2016). Aqueous phase reforming (APR) of glycerol over platinum supported on Al_2_O_3_ catalyst. Renew. Energy.

[56] Priya S.S., Kumar V.P., Kantam M.L., Bhargava S.K., Periasamy S., Chary K.V.R. (2015). Metal–acid bifunctional catalysts for selective hydrogenolysis of glycerol under atmospheric pressure: A highly selective route to produce propanols. Appl. Catal. A Gen..

[57] Gandarias I., Arias P.L., Requies J., Güemez M.B., Fierro J.L.G. (2010). Hydrogenolysis of glycerol to propanediols over a Pt/ASA catalyst: The role of acid and metal sites on product selectivity and the reaction mechanism. Appl. Catal. B Environ..

[58] Suprun W., Lutecki M., Haber T., Papp H. (2009). Acidic catalysts for the dehydration of glycerol: Activity and deactivation. J. Mol. Catal. A Chem..

[59] Du H., Chen S., Wang H., Lu J. (2017). Acidic alumina overcoating on platinum nanoparticles: Close metal–acid proximity enhances bifunctionality for glycerol hydrogenolysis. Chin. J. Catal..

[60] García-Fernández S., Gandarias I., Requies J., Soulimani F., Arias P.L., Weckhuysen B.M. (2017). The role of tungsten oxide in the selective hydrogenolysis of glycerol to 1,3-propanediol over Pt/WO_x_/Al_2_O_3_. Appl. Catal. B Environ..

[61] Liao X., Li K., Xiang X., Wang S.G., She X., Zhu Y., Li Y. (2012). Mediatory role of K, Cu and Mo over Ru/SiO_2_ catalysts for glycerol hydrogenolysis. J. Ind. Eng. Chem..

