# Supplementary material for: Influence of Boron, Tungsten and Molybdenum Modifiers on Zirconia Based Pt Catalyst for Glycerol Valorization
Source: Nanomaterials (Basel). 2019 Apr 2;9(4):509. doi: 10.3390/nano9040509 (PMC6523238; doi:10.3390/nano9040509)
Supplement: Supplementary file 1 [file nanomaterials-09-00509-s001.pdf]

Supplementary information

Influence of the modifiers on zirconia based Pt catalyst for glycerol valorisation

Manuel Checa, Vicente Montes, Jesús Hidalgo*, Alberto Marinas, and Francisco J. Urbano
Departamento de Química Orgánica, Universidad de Córdoba, Campus de Rabanales, Edf. Marie Curie (Anexo), E-14071 Córdoba, Spain
*Corresponding Author.
E-mail: jesus.hidalgo@uco.es

1- 2-propanol reaction test

Table S1. Propan-2-ol conversion and products distribution obtained in the gas-phase propan-2-ol transformation test reaction at 200°C (time on stream, 2h). Sel. Acid: selectivity to products formed on acid sites (propene and diisopropyl ether); Sel. Basic or Redox: selectivity to products formed on basic or redox sites (acetone and diacetone alcohol).

Catalyst	Conversion (%)	S. Propene (%)	S. D.Ether (%)	S. Acetone (%)	S. DA (%)
**ZrO_2**	0	0	0	0	0
**B/ZrO_2**	2.77	34.39	1.73	63.88	0
**Mo/ZrO_2**	61.66	79.32	2.54	18.14	0
**W/ZrO_2**	95.48	100	0	0	0
**$Pt//ZrO_2$-200**	100	5.69	0	66.05	25.42
**$Pt//B/ZrO_2$-200**	97.25	6.08	1.98	75.66	18.81
**$Pt//Mo/ZrO_2$-200**	97.22	17.89	27.94	43.39	0
**$Pt//W/ZrO_2$-200**	99.06	99.27	0	0.73	0
